# Recent Advances in Colorimetric Detection of Arsenic Using Metal-Based Nanoparticles

**DOI:** 10.3390/toxics9060143

**Published:** 2021-06-17

**Authors:** Haradhan Kolya, Kazuharu Hashitsume, Chun-Won Kang

**Affiliations:** 1 Department of Housing Environmental Design and Research Institute of Human Ecology, College of Human Ecology, Jeonbuk National University, Jeonju 54896, Jeonbuk, Korea; 2 Graduate Schools of Education, Shimane University 1060 Nishikawatsu-cho, Matsue, Shimane 690-8504, Japan; hashitsume@edu.shimane-u.ac.jp

**Keywords:** arsenic, nanoparticles, colorimetric detection, groundwater contamination, arsenicosis

## Abstract

Nowadays, arsenic (III) contamination of drinking water is a global issue. Laboratory and instrument-based techniques are typically used to detect arsenic in water, with an accuracy of 1 ppb. However, such detection methods require a laboratory-based environment, skilled labor, and additional costs for setup. As a result, several metal-based nanoparticles have been studied to prepare a cost-effective and straightforward detector for arsenic (III) ions. Among the developed strategies, colorimetric detection is one of the simplest methods to detect arsenic (III) in water. Several portable digital detection technologies make nanoparticle-based colorimetric detectors useful for on-site arsenic detection. The present review showcases several metal-based nanoparticles that can detect arsenic (III) colorimetrically at a concentration of ~0.12 ppb or lower in water. A literature survey suggests that biomolecule-based metal nanoparticles could serve as low-cost, facile, susceptible, and eco-friendly alternatives for detecting arsenic (III). This review also describes future directions, perspectives and challenges in developing this alternative technology, which will help us reach a new milestone in designing an effective arsenic detector for commercial use.

## 1. Introduction

Globally, intake of arsenic (III) and arsenic (V) ions via food and drinking water has dramatically increased, as per several recent reports [1,2,3,4,5,6,7,8]. Approximately 200 million people worldwide are affected by arsenic toxicity [9]. According to the World Health Organization (WHO), arsenic in drinking water at a concentration of >10 ppb is highly unsafe to community health [10,11]. Arsenic comes to the groundwater surface via magmatism and periodic erosion [12,13,14]. In addition, several human activities are also responsible for raising the concentration of arsenic levels in groundwater. Industries that discharge effluents with arsenic into the soil or natural water resources include those involved in agrochemicals, pesticides, wood processes, and preservatives [15,16,17].

In general, inorganic arsenite and arsenate salts are naturally present in groundwater. A small amount of arsenic comes from organic compounds, such as dimethyl arsenic acid, dithioarsenate, and monomethyl arsenic acid [18,19]. Arsenic may present in four oxidation states, e.g., −3, 0, +3, and +5. Out of these, the trivalent and pentavalent forms of arsenic species are harmful to animals and plants [20,21]. The toxicity may vary with the type of exposure and oxidation state of arsenic. In particular, trivalent arsenic species are more toxic than pentavalent arsenic species [9,22]. Long-term arsenic exposure causes a variety of diseases, including arsenicosis, hemolysis, cancer, neurological disorders, and painful patches on the hands and feet [20,23]. Therefore, arsenic (III) concentrations in drinking water should be measured to avoid any harm to living organisms. Many detection methodologies have been adopted to measure arsenic levels in water steam, such as Raman spectroscopy (RS) [24], the fontal chromatography–ICP–MS method (FC–ICP–MS) [25], total reflection X-ray fluorescence (TXRF) spectrometry [26], Surface-enhanced Raman spectroscopy (SERS) [27], electrothermal atomic absorption spectrometry (ETAAS) [28], inductively coupled plasma mass spectroscopy (ICP–MS) [29], laser-induced breakdown spectroscopy (LIBS) [30], and atomic fluorescence spectroscopy (AFS) [31], all of which are sufficient to detect arsenic. In addition, chemisorbent resins provide a new approach to arsenic speciation [32]. However, these instrument-based strategies require a managed lab environment, a long operating time, trained labor, and pure chemicals [33].

Moreover, instruments that require electrical power and additional services have restricted use for on-site applications [34]. Therefore, a cheap and facile method, such as colorimetric detection of arsenic in water, can be an effective alternative. recent decades, researchers have explored metal nanoparticles (alone or in combination) based on colorimetric detectors to overcome the sensing system’s drawbacks. Specifically, gold nanoparticles, silver nanoparticles, metal-organic frameworks (MOFs), and metal graphene nanocomposites are extensively employed to fabricate arsenic sensors [35,36,37,38,39,40,41,42]. These colorimetric sensors can detect arsenic (III) ions efficiently in an aqueous medium. Metal nanostructures have excellent properties for the colorimetric determination of arsenic. However, these need significant improvement for practical applications.

Therefore, this review work investigates the recent developments in metal-based nanoparticles for the colorimetric detection of arsenic (III) in water. This review summarizes the sustainable, cost-effective and efficient strategies for arsenic sensing to realize these sensors in the real world. 

## 2. Arsenic and Its Harmful Effects

**Figure 1:**
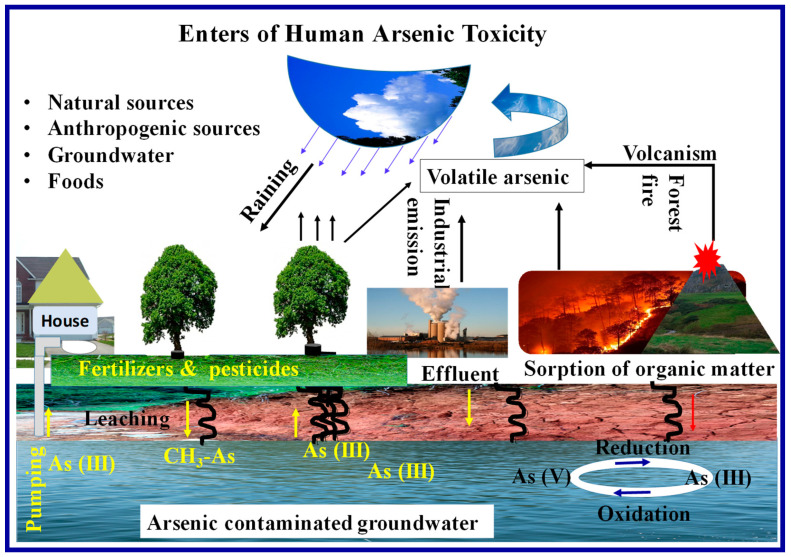
Schematic representation of human exposure to arsenic and arsenic cycle.

An arsenic metalloid is one of the most naturally abundant metalloids, and it is also the most carcinogenic metalloid to living organisms. Its isotope has four oxidation states, from −3 to +5. Water contamination with arsenic can occur through insecticides, industrial effluents, municipal sewage, and household waste [21,43]. A schematic representation of human exposure to arsenic is shown in Figure 1.

Daily intake of arsenic-contaminated drinking water can create acute to chronic health impacts. Acute arsenic toxicity has been reported to cause acute paralytic syndrome (APS) and acute gastrointestinal syndrome (AGS) [44]. The central nervous system is depressed, and the cardiovascular system collapses in APS. Central nervous depression is caused by the necrosis of both white blood cells (WBCs) and red blood cells (RBCs) [45]. Arsenic (III) affects blood vessels, causing blood circulation problems and the sensation of pins and needles in the hands and feet [44]. AGS symptoms start with a taste of garlic, burning lips, dry mouth, and dysphagia [46]. Adsorption of arsenic by human beings for a longer time results in black-foot illnesses [47], lung cancer [48], and bladder cancer [49]. The symptoms are initially insidious in arsenicosis and based on the dose magnitude and exposure duration [50]. The exact mechanism of the occurrence of arsenicosis diseases in human organs is unclear. Arsenic biomethylation is crucial for the elucidation of its toxic and carcinogenic action. Arsenic transforms enzymatically into methyl arsenic [CH_3_ AsO_2_^2−^] and dimethyl arsenic [(CH_3_)_2_ AsO_2_^2−^] [51]. Arsenic can also cause genetic changes such as inhibition of DNA repair enzymes and changes in DNA methylation patterns [52].

Specific skin patches in pigmentation and keratosis are the common characteristics of chronic arsenic toxicity. Pigmentation may also involve mucous membranes such as the tongue under the surface or buccal mucous membranes [50]. Additionally, Leucomelanosis tends to occur in a patient with arsenicosis [53]. Numerous epidemiological studies have examined the risk of various cancers associated with arsenic absorption through drinking water. Many of these studies are ecological, and others suffer from methodological faults, particularly in exposure measurement [54]. However, there is significant evidence that greater arsenic levels in drinking water are linked to cancer growth in various locations, including the skin, bladder, and lungs. Arsenic-induced illness, including cancer, is a significant public health issue across the globe [54]. Hence, it is urgent to measure the concentration of arsenic in drinking water to identify arsenic-contaminated water. The identification followed by purification of arsenic-contaminated water can control the arsenicosis disease.

## 3. Colorimetric Sensing of Arsenic

In terms of ease of signal transduction, colorimetric analysis of arsenic has become the most practically applicable method. The Gutzeit method is one of the most commonly used methods for colorimetric analyses of arsenic. This method was employed to develop arsenic field test kits [55]. Although the Gutzeit method-based technique is economical, it produces toxic arsine gas as by-products. The molybdenum blue is also frequently used to detect arsenic in water samples. The molybdenum blue-based method is specific to arsenic (V); the interaction between arsenic (V) and reduced molybdenum resulted in the appearance of the blue color [56]. Therefore, the molybdenum blue could differentiate arsenic (V) and arsenic (III).

Researchers explored metal nanostructure-based materials to make arsenic colorimetric sensors more sensitive, rapid, precise, economical, and efficient. Mainly, metal nanostructures-based sensors have been used to elaborate on a fundamental principle of color conversion, studied for colorimetric detection of arsenic solution [41]. This paper discusses the potential of metal-based nanoparticles for arsenic detection.

### 3.1. Gold-Based Nanoparticles

**Figure 2:**
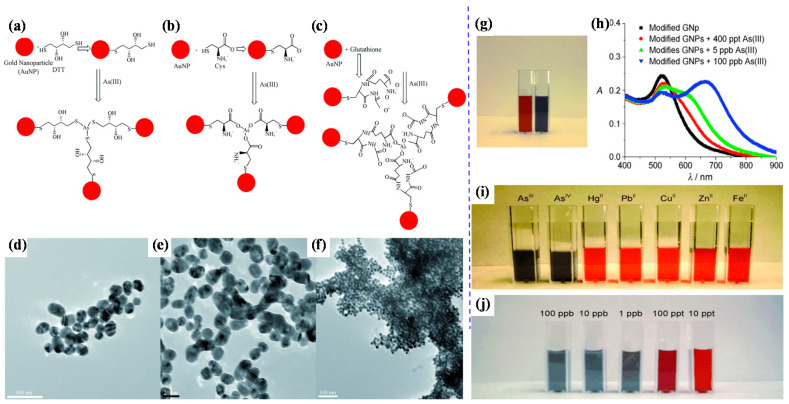
Representation of AuNP-based arsenic detection. (**a**) DTT-modified AuNPs; (**b**) Cys-modified AuNPs; (**c**) GSH-modified AuNPs; (**d**) TEM image showing GSH/DTT/Cys-modified AuNPs before the addition of arsenic (III); (**e**) TEM image demonstrating aggregation of GSH/DTT/Cys-modified AuNPs after addition of 80 ppb arsenic (III); (**f**) TEM image after the addition of 250 ppt arsenic (III); (**g**) photograph showing colorimetric change of GSH/DTT/Cys-modified gold nanoparticles upon addition of 800 ppb arsenic (III); (**h**) absorption profiles of modified gold nanoparticles before and after addition of arsenic (III) ions; (**i**) photograph showing colorimetric changes of GSH/DTT/Cys-modified gold nanoparticles in the presence of PDCA upon addition of various metal ions (5 ppb) and (**j**) different concentrations of arsenic (III) **[61]**. Copyright 2009, reproduced with permission from WILEY-VCH Verlag GmbH & Co. KGaA, Weinheim, Germany.

Scientists have focused extensively on developing gold nanoparticle (AuNP)-based sensors to detect arsenic in water samples. Recently, gold-modified lauryl sulfate nanoparticles with a limit of detection (LOD) of 2 ppb were reported for colorimetric sensing of arsenic (III), using localized surface plasmon resonance (LSPR) [57]. The LSPR band shifted due to the color change of AuNPs—i.e., pink to blue—with arsenic (III) ions due to the inter-particle coupling effect. Lauryl sulfate acts as a capping agent of AuNPs and is aggregated and replaced by the arsenic contaminant. The modification of AuNP surfaces with sulfur-containing compounds is highly beneficial in enhancing AuNP-based colorimetric sensors’ sensitivity; arsenic generally displays the intrinsic property of a potent binding affinity for sulfur-containing compounds. Therefore, glutathione (GSH), dithiothreitol (DTT), cysteine (Cys), and 2,6-pyridine dicarboxylic acid (PDCA) [GSH-DTT-CYs-PDCA]-functionalized AuNPs can detect arsenic (III) in water [58]. Arsenic (III) has a strong affinity for these ligands [59,60]. Arsenic (III) ions can interact with 3 DTT-conjugated gold nanoparticles through an As-S bond, as shown in Figure 2A–F [61]. However, there is no free SH group available for binding with arsenic (III) ions in the case of GSH- or Cys-conjugated gold nanoparticles. Figure 2G,H shows the colorimetric response of GSH/DTT/Cys-modified gold nanoparticles after the addition of arsenic (III) [61]. The addition of PDCA improved the test selectivity for arsenic (III) ions much more because PDCA could not interact with gold nanoparticles through the SH linkage in the same way as DTT, GSH, and Cys (Figure 2I,J) [61].

Moreover, it exhibited a LOD of 1 ppb, which is less than the allowable limit (as per Environmental Protection Agency (EPA) guidelines) of arsenic. Eco-friendly glucose-functionalized gold nanoparticles are also sufficient for the colorimetric detection of arsenic (III) in water [41]. The glucose-functionalized AuNPs exhibited an LOD of 0.53 ppb. Hydroxyl groups of glucose interacted with gold particles and formed chemical bonds, stabilising gold nanoparticles and reducing the inter-particle distance among the nanoparticles. The color of nanoparticles changes depending on their inter-particle distance. Glucose-functionalized gold nanoparticles showed a red color, but this changed sharply to blue with arsenic [41].

**Figure 3:**
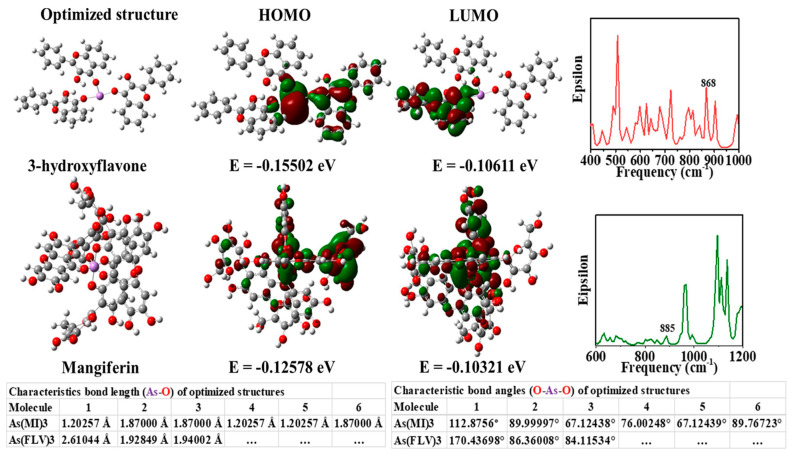
Images of theoretically optimized arsenic complexes with FLV (3-hydroxy flavone) and mangiferin (MI), highest occupied molecular orbital (HOMO), lowest unoccupied molecular orbital (LUMO), and a theoretical infrared (IR) spectrum [64]. Copyright 2020, reproduced with permission from Elsevier B.V., Amsterdam, The Netherlands.

Moreover, citrate-capped gold nanoparticles showed a detection limit for arsenic (III) ions that was lower than 10 ppb due to more interaction of citrates ion with arsenic (III) ions [62]. Additionally, *Mangifera indica* Leaf Extract mediated AuNPs can detect arsenic at a limit of 1.2 ppb by the colorimetric detection technique. The leaf extract of the *Mangifera indica* acted as a reducing and stabilizing agent [63]. Encapsulation of gold *Mangifera indica* flower extract can detect arsenic (III) ions in water at optimum conditions [64]. Using LC–MS/MS, the authors reported that *Mangifera indica* flower extract contains more mangiferin (977 ppb) than 3-hydroxy flavone (4 ppb). As shown in Figure 3, the theoretical study shows that the mangiferin and 3-hydroxy flavone present in *Mangifera indica* flower extract are responsible for detecting arsenic in aqueous media [64].

**Figure 4:**
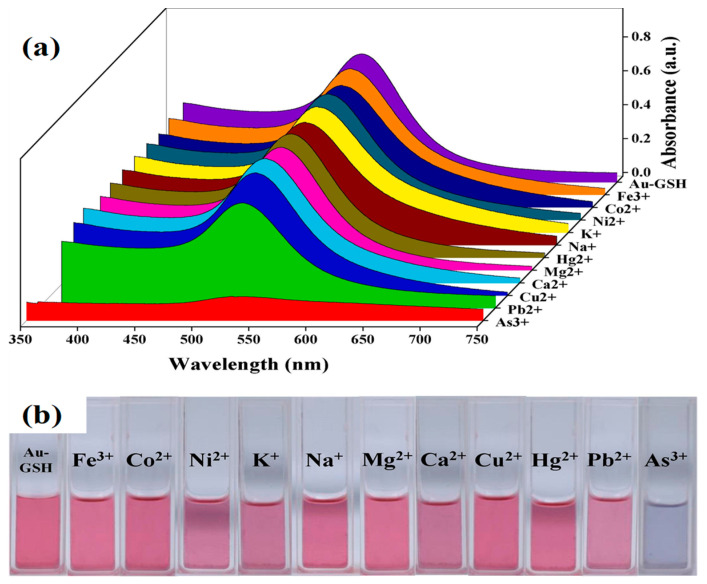
(**a**) Absorption spectra of GSH-AuNPs in the presence of different metallic ions and (**b**) the digital photograph of color changes of GSH-AuNPs in the presence of different metallic ions at a concentration of 10 ppb and while arsenic (III) was 1 ppb [65]. Copyright 2020, reproduced with permission from Elsevier B.V., Amsterdam, The Netherlands.

However, glutathione-functionalized gold nanoparticles in RGB can carry out a fast colorimetric detection of arsenic (III) [65]. The detection limit of arsenic was 0.12 ppb, with a detection accuracy of around 2%. As shown in Figure 4, GSH-functionalized AuNPs displayed excellent selectivity towards arsenic (III) ion in a water medium. Arsenic ions bind to GSH ligands, causing AuNP aggregation and a rapid color change in the solution [65].

### 3.2. Silver-Based Nanoparticles

Silver nanoparticles provide a rapid response to localized surface plasmon resonance compared to gold nanoparticles with enhanced sensitivity [66]. As in AuNPs, various capping agents have been exploited to construct silver nanoparticles for sensitive and selective sensing of arsenic. PEG-functionalized silver nanoparticles’ are well-suited for detecting arsenic (III) ions in an aqueous medium [40]. The PEG-modified silver nanoparticles are sufficient enough to detect arsenic (III) in 1 ppb due to the addition of PEG. In addition, PEG-functionalized silver nanoparticles have adjustable negative surface charges, responsible for the stability of nanoparticles, and the electrostatic repulsion between negatively charged surfaces of silver nanoparticle protects them from accumulation.

**Figure 5:**
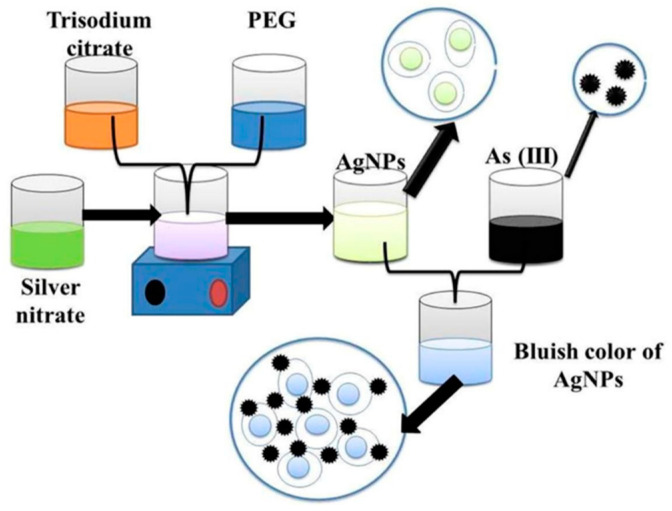
Functionalized silver nanoparticles as an effective medium towards trace determination of arsenic (III) in aqueous solution [40]. Copyright 2019, reproduced with permission from Elsevier B.V., Amsterdam, The Netherlands.

Interestingly, in the presence of arsenic (III), these functional silver nanoparticles interacted with PEG hydroxyl groups, which led to the aggregation of silver nanoparticles [67]. As a result, the color of functionalized nanoparticles changed from yellow to bluish [40], as shown in Figure 5. Additionally, arsenic in Aptamer-AgNP solution remarkably decreases the absorbance peak due to the formation of the As–Aptamer–AgNPs complex. This testing method indicates highly selective detection of arsenic (III) ions with a LOD of 6 ppb and a linear range of 50 to 700 ppb [68].

**Figure 6:**
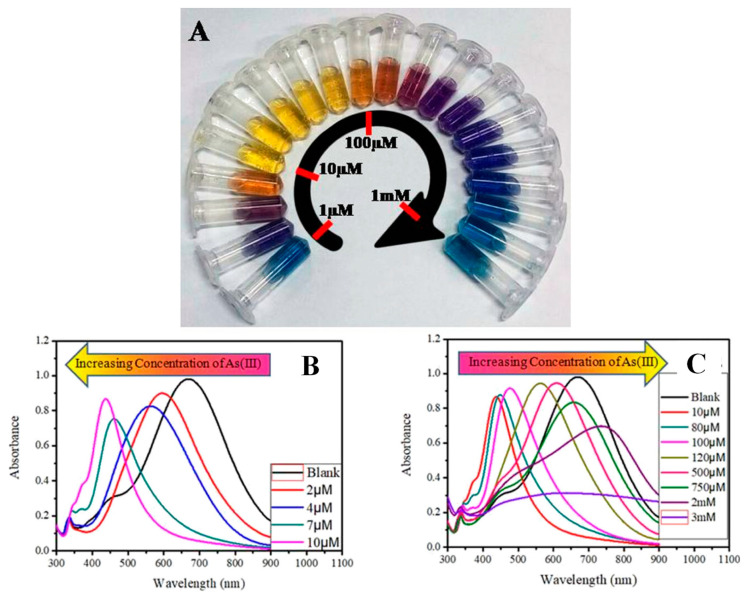
(**A**) Concentration-dependent color-coded sensing of arsenic (III) between the concentration range of 10^−6^ to 10^−3^ M, (**B**) tuning of SPR as a result of morphological change of AgNPr at different concentrations of arsenic (III) between 10^−6^ and 10^−3^ M where (**B**) shows the variation of plasmon band at different lower concentrations of arsenic (III) in the range of 0.0–10.0 μM (0.0 μM (blank): black trace (λmax = 704 nm), 1.0–2.0 μM: blue trace, 2.0–4.0 μM: orange trace, 5.0–7.0 μM: red-violet trace, 8.0–10.0 μM: blue-violet trace) and (**C**) at different higher concentrations of arsenic (III). The plasmon band, and hence the color of the nanomaterials, changes in a distinct manner, where a specific color remains unchanged in a broader range of growing concentrations such as: 10.0–80.0 μM: yellow, 90.0–100.0 μM: orange, 110.0–200.0 μM: dark red, 250.0–500.0 μM: purple, 750.0 μM to 2 mM: different shades of blue, 3–10 mM: faded blue, and above 10 mM the color becomes faint blue to grey or almost colorless [69]. Copyright 2019, reproduced with permission from American Chemical Society, Washington, DC, USA.

AgNPs functionalized with polyvinylpyrrolidone (PVP) have a significant affinity for arsenic (III) ions, as adding arsenic (III) ions to PVP-AgNP improved electrostatic interactions and morphological changes in nanoparticles. The UV–Vis spectra of AgNPs with different concentrations of arsenic (III) ions are shown in Figure 6 [69]. In addition, silver nanoplates (AgNPls) changed color quickly in the presence of arsenic (III) and arsenic (V). Ferrihydrite-coated silica gel has improved the selectivity of AgNPs towards arsenic (V) (SiO_2_-Fh). The AgNP-SiO_2_-Fh Acomposites can detect arsenic in concentrations ranging from 500 to 30000 ppb [70].

Recently, multi-ligand-based AgNPs were studied to detect arsenic (III) using the colorimetric approach. It could be synthesized by the chemical reduction method using asparagine (Asn) as the capping agent and further alteration with reduced GSH and DTT. The synthesized GSH/DTT/Asn–AgNPs could be used as multifunctional probes for an multimodal arsenic assay (III) due to their outstanding plasmonic properties and characteristic electrochemical activity. This approach can detect arsenic even at a low concentration of 0.36 ppb [71].

### 3.3. Metal Oxide-Based Nanoparticles

**Figure 7:**
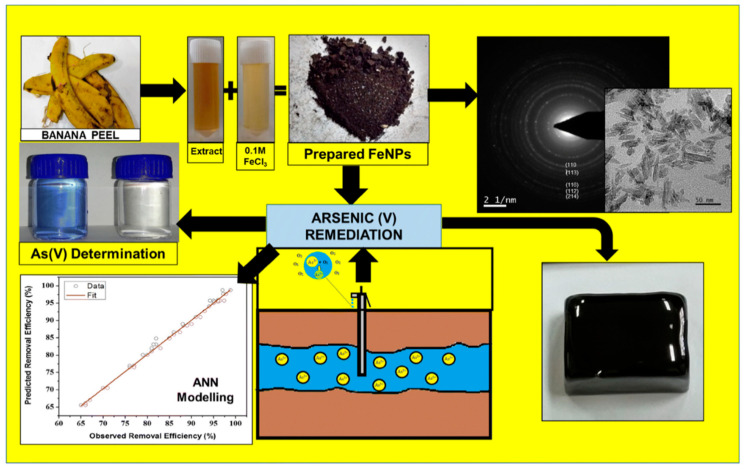
A schematic representation of the synthesis of Fe_3_O_4_ and detection of arsenic [74]. Copyright 2019, reproduced with permission from Springer-Verlag GmbH, Heidelberg, Germany.

Nanostructured transition metal oxides such as Fe_3_O_4_, MgO, TiO_2_, ZnO, NiO, SnO_2_, CeO_2_, MnO_2_, ZrO_4_, and NiWO_4_ are used for heavy metal sensing. Transition metals are usually economical, highly conductive, suitable adsorbents and highly stable [72]. Therefore, metal nanoparticles displayed excellent performances for the detection of arsenic (III) ions in water. For example, Fe_3_O_4_ nanoparticles bonded with gold ligands exhibited excellent selectivity and quick visual detection of arsenic. The Fe_3_O_4_ @Au-based colorimetric system exhibited a LOD of 0.86 ppb for arsenic (III) detection [73]. In another report, α-Fe_2_O_3_ was prepared from a waste banana peel extract because banana peel contains excessive polyphenols and flavonoids that act as reducing agents. Almost similar size (60 nm) nanoparticles were used to simultaneously detect and adsorb arsenic (V). The α-Fe_2_O_3_-based colorimetric sensor exhibited a LOD of 100 ppb for arsenic (V) [74]. The positive charge of nanoparticles facilitated the high adsorption of negatively charged arsenate ions due to electrostatic interaction. A schematic of the synthesis of α-Fe_2_O_3_ and its application in detecting arsenic (V) is shown in Figure 7 [74].

**Figure 8:**
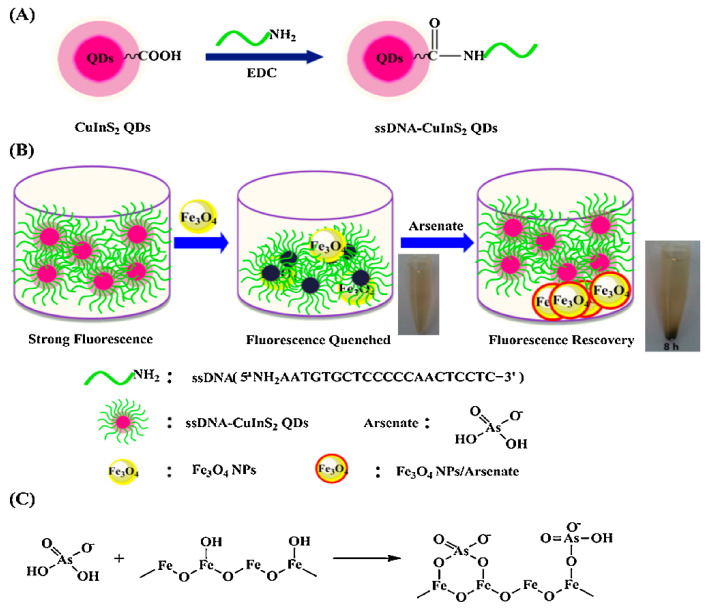
(**A**) Schematic illustration for the fabrication of ssDNA-CuInS_2_ QDs. (**B**) The schematic illustration of sensing arsenate by ssDNA-CuInS2 QDs@Fe_3_O_4_ NPs and the arsenate removal photographs by a magnet. (C) Schematic illustration of the mechanism of arsenate adsorption onto Fe_3_O_4_ NPs [77]. Copyright 2015, reproduced with permission from Elsevier B.V., Amsterdam, The Netherlands.

DNA-functionalized Fe_3_O_4_ nanoparticles showed significant affinity and selectivity towards arsenic and could be used to detect arsenic in water up to 0.95 ppb by the fluorescence quenching technique [75]. Furthermore, other nanoparticles, e.g., CeO_2_ nanoparticles, were modified with DNA to investigate arsenic levels [76]. The desorption of DNA from nanomaterials is caused by interactions between DNA-conjugated nanostructures and arsenic. The results showed that CeO_2_ nanoparticles had improved performances compared to Fe_3_O_4_, with the LOD nearly 10-fold less than Fe_3_O_4_ [76]. A novel CuInS_2_ quantum dots@magnetic Fe_3_O_4_ nanocomposite-based "turn off" nanosensor for arsenic detection was revealed. The CuInS_2_ quantum dots@magnetic Fe_3_O_4_ was able to detect at as low as 10 ppb [77]. A schematic illustration of the fabrication of CuInS_2_ quantum dots@magnetic Fe_3_O_4_ is shown in Figure 8.

Cobalt oxyhydroxide (CoOOH) nanoflakes showed significant arsenic detection efficiency in addition to iron oxide [78]. CoOOH nanoflakes show peroxidase-like activity, which produces a green-colored oxidation product in the presence of H_2_O_2_ and 2,2′-azinobis (3-ethylbenzothiazoline-6-sulfonic acid) (ABTS). Interestingly, the green color was not observed in the presence of arsenic, as arsenic binds with CoOOH through electrostatic attraction and forms an As–O bond to inhibit peroxidase-like activity. Therefore, it can effectively detect arsenic in water using the colorimetric method with a LOD of 3.72 ppb [78].

### 3.4. Metal GO- or CNT-Based Nanoparticles

A variety of nano-scale carbon-based building blocks, including nanotubes, graphene and graphene oxide, have drawn significant interest as electrode materials for detection of heavy metals owing to their extraordinary physical and chemical properties, i.e., elevated surface area, high electrical conductivity, powerful mechanical strength, biocompatibility and low manufacturing costs [79,80]. Graphene oxide (GO) has a two-dimensional plane and many functional groups containing oxygen with the disorder on the basal planes and edges. The GO develop significant mechanical properties and chemical sensing activity [81]. Recently, a magnetic graphene quantum dot-based sensor (fluorescence probe) was reported as a highly sensitive and arsenic-selective material [82]. The fluorescent zinc oxide and CdS quantum dots (QDs) were revealed as arsenic sensor components by fluorescence spectroscopy [83,84]. A magnetic graphene quantum dot-based sensor yielded better outcomes than ZnO (QDs) and CDS (QDs) due to the presence of iron oxide, which offered more contact for the formation of the chelating complex with arsenic in the medium [82]. A research group has recently reported a highly selective and sensitive and cost-effective prism-based SPR sensor integrated with a hydrous ferric oxide-magnetite-reduced graphene oxide nanocomposite to detect arsenic ions at a detection limit of 0.1 ppb [85].

### 3.5. Metal-Organic Framework

**Figure 9:**
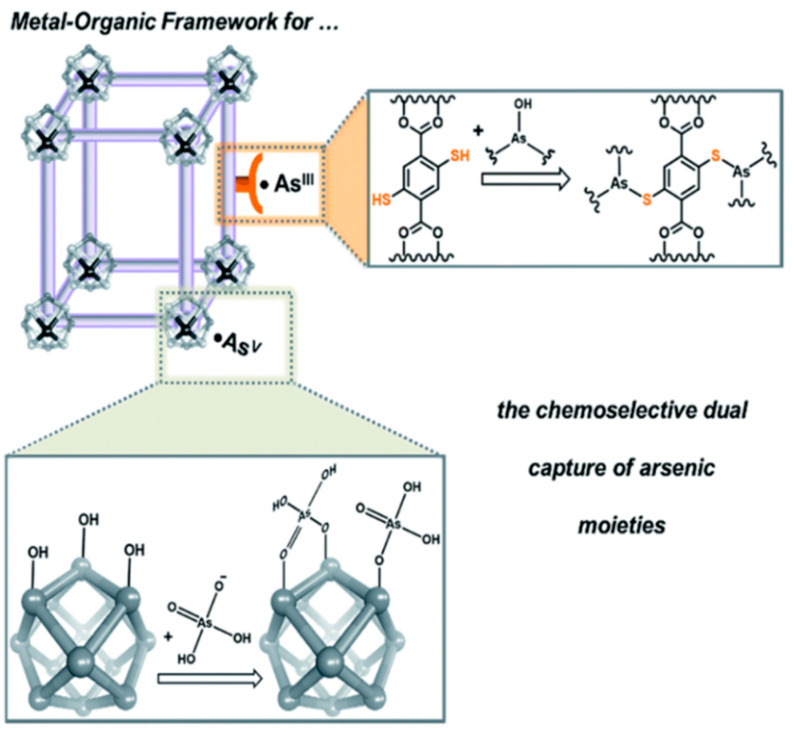
A schematic illustration illustrating how MOFs can be tailored to coordinate anionic arsenic (V) moieties at the node while binding neutral arsenic (III) to the framework [90]. Copyright 2016, reproduced with permission from Royal Society of Chemistry, London, U.K.

The MOFs are essential in separation, drug delivery and catalysis fields [86]. The MOF shows the attractive hybrid characteristic of organic bridging ligands and metal particulates in a framework that displays a larger surface area [87]. The large surface area of MOFs offers multiple channels for guest molecules to enter and interact with the framework. This phenomenon is quite helpful for trapping targeted pollutants effectively and fulfils the requirement of detection and removal of contaminates. The intrinsic open pore structure and extensive channels can encourage the quick diffusion and transportation of targeted pollutants, thus ensuring a rapid response time, selective detection and fast kinetics [88]. Hence, MOF materials are becoming promising candidates for sensing and removing arsenic simultaneously [89]. Several recent studies have shown MOFs’ proper function and composites to detect and remove arsenic [90,91]. Figure 9 illustrates a modification of MOFs to coordinate arsenic (V) moieties at the node [90]. Therefore, amino-decorated MOF products are interesting.

The amino-functionalized iron-based MOFs showed good selectivity for arsenic (III) identification. The most frequently observed MOF structure warped after coming into contact with water. High-valance metal ions such as Cr (III), Zr (IV), Fe (III), and Al (III) were used to build chemically stable coordination bonds to improve the water stability of ligand-based carboxylate MOF. The introduction of ligands with hydrophobic functionality such as methyl, ethyl, and trifluoromethyl is important to protect metal bodies from hydrolysis [92,93]. Therefore, two organic tritopic carboxylic acids with methyl and ethyl groups, dimethyl-5′-(4(methoxycarbonyl)phenyl)-2′,4′,6′-trimethyl-[1,1′:3′,1′′-terphenyl]-4,4′′-dicarboxylic acid (H3CTTA) and dimethyl-2′,4′,6′-triethyl-5′-(4-(methoxycarbonyl) phenyl)-[1,1′,3’,1′′-terphenyl] -4,4′′-dicarboxylic acid (H3CETA) were synthesized. Then, both H3CTTA and H3CETA were reacted with aluminium nitrate in DMF solvent, respectively. The materials derived from this reaction were observed to be extremely porous and labeled Al-MOF (CTTA) and Al-MOF (CETA). Al-MOF (CTTA) exhibited an improved detection efficiency to arsenic (III) from roxarsone (ROX) and nitrosone (NIT) [92].

The above information has been summarized in Table 1 to compare the colorimetric detection performances of nanoparticles.

### 3.6. Future Directions, Perspectives and Challenges

Despite the recent advances in this area, there are still many issues to be addressed.

(1) pH and temperature are significant for detecting arsenic (III) ions in water. There are only a few colorimetric methods for detecting arsenic (III) with the variation of temperature and pH. Therefore, the development of new nanomaterials would support their practical applications.

(2) Although some reported nanomaterials have good sensitivity, they are often affected by other metal ions found in groundwater. Therefore, research and development on the sensitivity of nanomaterials should be sufficient to detect arsenic in other situations that are also of great interest.

(3) Some biomolecule-based nanoparticles have higher sensitivities, but they are challenging to use in practice due to the reproducibility of steady-size nanoparticles. Since the size of the nanoparticles is the key in the detection of arsenic (III), research and development on repeated synthesis with regular-sized nanoparticles is still of the utmost importance.

(4) Further research on arsenic (III) detection using metal nanoparticles will be necessary to enhance practical application.

(5) Nanoparticles should be designed to be low-cost, simple to use, environmentally friendly, and have practical applicability accessible to the general public.

Future research in this field should focus on developing novel highly selective and sensitive nanoparticles for colorimetric detection of arsenic that are simple to use, anti-interference, fast, have a low detection limit and environmentally friendly.

## 4. Conclusions

This review highlights metal nanoparticles’ progress for detecting arsenic in aqueous media by colorimetric techniques. Compared to other techniques, the colorimetric methods covered in the present review showed better sensing efficiency. Here, we discussed the colorimetric detection of arsenic (III) in comparison to AuNPS, AgNPs, metal oxide nanoparticles, metal GO or CNT-based materials, and metal-organic frameworks. We found that colorimetric methods for detecting arsenic with various AuNPs were appealing. This paper could help to develop new nanomaterials for colorimetric detection of arsenic (III).

## Figures and Tables

**Table 1:** The LOD of arsenic (III) using various nanoparticles and a colorimetric approach.

No	Metal Nanoparticles	Limit of Detection (ppb)	Range of Detection (ppb)	Reference
1.	S–layer protein–AuNPs	240	240–2400	[37]
2.	Glucose–AuNPs	0.53	1–14	[41]
3.	AuNPs-lauryl sulfate	2.0	5–500	[57]
4.	GSH–DTT-CYs–PDCA–AuNPs	2.5	2–20	[58]
5.	Glutathione + AuNPs	0.003	n.a.	[61]
6.	AuNPs-PEG	5.0	n.a.	[94]
7.	Aptamer-based AuNPs	1.26	1.26–200	[95]
8.	Aptamers-AuNPs-surfactant	0.6	1–1500	[96]
9.	Citrate-capped AuNPs	1.8	4–100	[62]
10.	*Mangifera indica* leaf extract–AuNPs	1.2	n.a.	[63]
11.	GSH-functionalized AuNPs	0.12	n.a.	[65]
12.	ssDNA–AuNPs	0.18	1–30	[97]
13.	Aptamer–CTAB–AuNPs	16.9	1–100	[98]
14.	AuNPs DNA aptamer	161	76.6–766	[99]
15.	DMSA-Au nanorod	1.0	n.a.	[100]
16.	DTT–AuNRs	10	10–100.1	[101]
17.	Europium–AuNPs	10	n.a.	[102]
18.	Au-cationic polymer and aptamer	5.3	n.a.	[103]
19.	Peptide–AuNPs	1.5	n.a.	[104]
20.	Thioctic acid–thioguanine–AuNPs	1.0	n.a.	[105]
21.	Asparagine–AuNPs	100	100–2000	[106]
22.	Sucrose–AuNPs	20	50–3000	[107]
23.	PEG–AgNPs	1.0	5–13	[40]
24.	Aptamer–AgNPs	6.0	50–700	[68]
25.	AgNPls-SiO_2_-Fh	500	500–3000	[70]
26.	AgNPls-SiO_2_-Fh	500	500–30,000	[70]
27.	GSH/DTT/Asn–AgNPs	0.36	0.4–20	[71]
28.	Fe_3_O_4_ (core)-gold (shell)-thiol ligands	0.86	n.a.	[73]
29.	α-Fe_2_O_3_	100	100–2000	[74]
30.	DNA-functionalized Fe_3_O_4_ nanoparticles	0.95	n.a.	[75]
31.	CuInS_2_ quantum dots@magnetic Fe_3_O_4_	10	0.015–15384.6	[77]
32.	Cobalt oxyhydroxide (CoOOH) nanoflakes	3.72	4–500	[78]
33.	Zinc oxide modified with curcumin	100	100–3000	[108]
34.	Oxidase-mimicking activity of Mn_3_O_4_ NPs	1320	5000–100,000	[109]
35.	Dithiothreitol-capped Pd nanoparticles	3.5	3.3–333,330	[110]
36.	Hemin-H_2_O_2_	6	10–200	[111]
37.	L-arginine-modified FeOOH	420	670–3,333,330	[112]

n.a. @ represents core and shell.

## Data Availability

Not applicable.

## Abbreviations

AFS	Atomic Fluorescence Spectroscopy
AgNPls	Silver Nanoplates
AgNPs	Silver Nanoparticles
AGS	Acute Gastrointestinal Syndrome
APS	Acute Paralytic Syndrome
AuNPs	Gold Nanoparticles
CNT	Carbon Nanotube
Cys	Cysteine
LOD	Limit of detection
DMSA	Meso-2,3-Dimercaptosuccinic Acid
DNA	Deoxyribose Nucleic Acid
DTT	Dithiothreitol
EPA	Environmental Protection Agency
ETAAS	Electrothermal atomic absorption spectrometry
GO	Graphene Oxide
GSH	Glutathione
ICP-MS	Inductively Coupled Plasma Mass Spectroscopy
LIBS	Laser-Induced Breakdown Spectroscopy
LSPR	Localized Surface Plasmon Resonance
MOF	Metal-Organic Framework
PDCA	Pyridine Dicarboxylic Acid
PEG	Polyethylene Glycol
ppb	Parts Per Billion
PVP	Polyvinylpyrrolidone
QDs	Quantum Dots
RBC	Red Blood Cell
RS	Raman Spectroscopy
WBC	White Blood Cell
